# Influence of high-definition transcranial direct current stimulation to the parietal cortex on postural control: a single-blind randomized crossover study

**DOI:** 10.3389/fnhum.2025.1546631

**Published:** 2025-05-26

**Authors:** Fan Yang, Yang Liu, Xianglin Lv, Yaqi He, Jinpeng Gao, Pei Zhang, Qing Li, Zhongmei Peng, Jinghua Qian

**Affiliations:** ^1^School of Sports Medicine and Rehabilitation, Beijing Sport University, Beijing, China; ^2^Key Laboratory of Exercise Rehabilitation Science of the Ministry of Education, Beijing Sport University, Beijing, China

**Keywords:** postural control, postural response, transcranial direct current stimulation, parietal lobe, sensory information processing

## Abstract

**Background:**

The parietal lobe is an important cerebral cortex area for sensory information processing to maintain postural control. High-definition transcranial direct current stimulation (HD-tDCS) can improve the excitability of the target brain region. The purpose of this study was to investigate whether HD-tDCS applied to either unilateral or bilateral parietal lobes would improve postural control.

**Method:**

A single-blind randomized crossover experimental design was used. 18 healthy right-handed adults were recruited for unilateral and bilateral HD-tDCS, as well as sham stimulation. All participants completed the sensory organization test (SOT) and motor control test (MCT) under eyes open and eyes closed conditions before and immediately after each intervention. The equilibrium score (ES), composite score (CS), and sensory score (VIS, SOM, VEST, PREF) from SOT, along with latency and response strength from the MCT, were calculated. Two-way repeated measures analyses of variance (ANOVAs) were used for the dependent variables. Bonferroni’s *post hoc* tests were used in case of significant ANOVA results.

**Results:**

The composite latency increased significantly after right (*p* = 0.025) and bilateral (*p* = 0.004) stimulation under eyes open condition. When the balance plate moved large forward, the latency increased significantly after left (*p* = 0.003) and bilateral (*p* = 0.04) stimulation under eyes closed condition. For response strength, when the balance plate moved forward at different magnitude under eyes closed condition, they all decreased significantly after bilateral stimulation (*p <* 0.05).

**Conclusion:**

The parietal lobe participates in the modulation of automatic postural response. The primary function of the right parietal lobe in postural response is to process visual information, while the left is responsible for processing somatosensory information.

## 1 Introduction

Sensory information processing are one of the important factors to maintain stability. The integration of sensory information from the somatosensory, visual, and vestibular systems serves to elucidate the complex sensory environment. The dependence of visual, somatosensory, and vestibular sensory cues is dynamically adjusted based on the particular environment, organism, or task conditions (Legrand et al., 2024). When equilibrium is disrupted, the central nervous system (CNS) reprocesses sensory information, selects and initiates appropriate postural responses, thus formulating an effective motor strategy to preserve stability (Slijper and Latash, 2004).

Postural control involves multiple regions of CNS, with the parietal lobe being a significant brain region for processing sensory information (Kamada and Takeuchi, 2023). Research have confirmed that the right parietal lobe area can be activated by visual stimuli (Naito et al., 2003), while the left can be stimulated by somatosensory and vestibular inputs (Ishigaki et al., 2016b). The right parietal lobe area is responsible for maintaining static stability through processing visual information, while the left processes proprioceptive information for the same purpose (Oka et al., 2022). Sensory information processing and integration in the posterior parietal cortex have been shown to be dominated by the right hemisphere (Arshad et al., 2014; Pisella et al., 2011; Convento et al., 2018). Therefore, it suggests that there are functional differences in sensory information processing between the left and right hemispheres of the parietal lobe.

Transcranial direct current stimulation (tDCS) is a non-invasive brain stimulation technique that delivers weak electrical currents to specific brain regions through the scalp. By inducing depolarization or hyperpolarization of neural tissues, it modulates cortical excitability and promotes changes in neural plasticity. Specifically, anodal stimulation increases neuronal excitability in targeted areas, while cathodal stimulation exerts an inhibitory effect, reducing cortical activity (Geers et al., 2024; Kesikburun, 2022). High-definition transcranial direct current stimulation (HD-tDCS) is capable of simultaneously modulating multiple regions within a distributed brain network (Müller et al., 2022). Research has demonstrated that HD-tDCS achieves focal stimulation by utilizing smaller electrodes arranged in a ring configuration, which effectively confines current flow to the targeted area (Kim et al., 2024). Therefore, an increasing number of studies have applied HD-tDCS to relevant brain regions to explore its impact on postural control, but few have approached it from the perspective of sensory information processing.

The weighting of reliance on sensory information (somatosensory, vision, and vestibular input) during postural control varies. An over-reliance on a specific sensory modality may result from either diminished quality of alternative sensory inputs or compromised neural processing of other sensory information within the brain. Research indicates that certain populations, such as individuals with chronic low back pain (Zafar et al., 2024), adolescents diagnosed with idiopathic scoliosis (Lau et al., 2023), patients recovering from ACL injuries (Ciceklidag et al., 2024), elderly individuals and those at high risk of falls (Unver and Bek, 2022), demonstrate compromised ankle joint proprioceptive integration capabilities. Patients with stroke (Moore et al., 2024) and Parkinson’s disease (Lee et al., 2025) exhibit an over-reliance on visual information for postural control. This compensatory strategy results in a predominant dependence on visual cues, which may include inappropriate or misleading inputs, while underutilizing vestibular and somatosensory feedback mechanisms. If unilateral or bilateral parietal HD-tDCS can optimize sensory information processing—with right-sided stimulation enhancing the visual system and left-sided stimulation enhancing the somatosensory system to maintain stability—it may offer a promising approach to modulate the aforementioned conditions and support the development of more targeted rehabilitation strategies.

Therefore, the primary purpose of this study was to investigate whether HD-tDCS applied to the left, right or bilateral parietal lobes enhances posture control and response, as well as the capacity of visual, somatosensory and vestibular system to maintain stability. The hypothesis of this study was that HD-tDCS of the unilateral and bilateral parietal lobes could improve postural control. The anodal tDCS applied on the right lobe could promote the ability of visual system, and applied on the left lobe could promote the ability of somatosensory system to maintain stability.

## 2 Materials and methods

This experiment employed a single-blind randomized crossover experimental method and was approved by Sports Science Experiment Ethics Committee of Beijing Sport University (No. 2023179H). Additionally, it obtained the Chinese clinical trial registration number: ChiCTR2300075357.^1^ All participants signed informed consent in accordance with the Declaration of Helsinki.

### 2.1 Participants

A total of 18 right-handed healthy young adults aged 18–35 years participated in this study [male, *n =* 8; female, *n* = 10; age, 21.88 (1.96); height, 1.71 (0.07); weight, 64.97 (9.83)]. According to G-power (effect size, 0.25; 1-*β*, 0.9; *α*, 0.05), 16 subjects needed to be recruited. To mitigate subject attrition, a total of 18 participants were recruited, all of whom completed the experimental procedures. Health status was assessed using Physical Activity Readiness Questionnaire (PAR-Q), while right-handedness was determined using the Edinburgh Handedness Inventory. Subjects with a history of neurological, orthopedic or other medical diseases were excluded.

### 2.2 HD-tDCS

**Figure 1:**
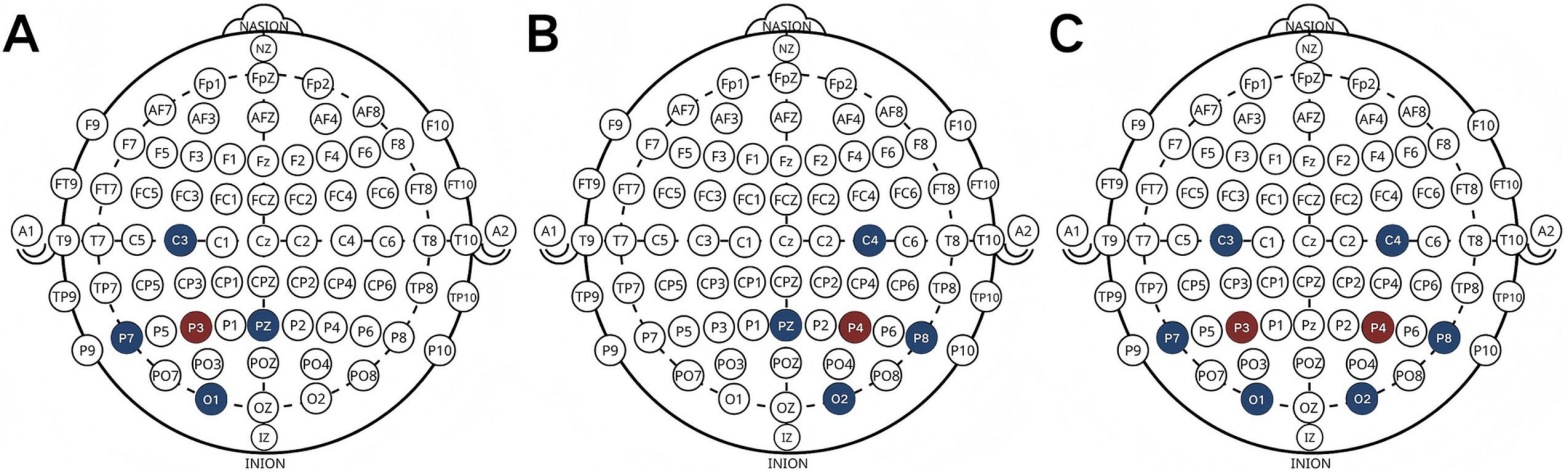
Electrode placement [left **(A)**, right **(B)** and bilateral **(C)**].

HD-tDCS and sham stimulation were delivered using a battery-driven current stimulator (NE, Starstim 32, Spain). A multifocal HD-tDCS montage was arranged in a 4×1 arrangement across unilateral hemispheres, utilizing five round Ag/AgCl electrodes measuring 3.14cm^2^. The central electrodes, using the anode, were positioned over the right (P4) or left (P3) parietal areas, with the cathodes serving as surrounding return electrodes (C4, P8, PZ, O2 on the right and C3, P7, PZ, O1on the left). When stimulating both hemispheres bilaterally, eight AgCl electrodes (3.14cm^2^ each) were used, with the central electrode positioned to stimulate the right (P4) and left (P3) parietal lobes simultaneously. To minimize errors arising from electrode variations, the return electrodes were positioned at P7, P8, C3, C4, O1, and O2. Each anode receives a current intensity of 2 mA, resulting in individual return electrodes receiving 0.50 mA for unilateral stimulation and 0.67 mA for bilateral stimulation. This study primarily investigates the functional disparities between the left and right parietal lobes, necessitating the assurance that each hemisphere receives equivalent intensity during each intervention. Stimulation lasted 20 min with 30s ramp up and ramp down periods. In the sham stimulation group, current was only passed during the ramp-up and ramp-down periods (Figure 1).

### 2.3 Measures

All subjects completed sensory organization test (SOT) and motor control test (MCT) on the Bertec Balance Advantage System (Columbus, U.S.A). The participants stood barefoot on the platform with their hands naturally hanging at their sides, and were instructed to gaze straight ahead and maintain standing stability to the best of their ability. Throughout the entire test, participants wore a harness, which did not impede their movements but served to ensure safety in the event of balance loss. In the event of the participants experiencing a loss of balance, the re-measurement was conducted.

SOT quantifies the ability of the sensory system (including visual, somatosensory and vestibular system) to maintain balance by inputting various sensory information conditions. This test consists of six conditions. Within each SOT, participants underwent three successive trials for each of the six conditions. Each trial lasted for 20 s, with a 10-s break between trials.

MCT assesses the latency and response strength of automatic postural responses when the platform moves forward or backward at three different magnitudes (small, medium, and large). The participants were tested under conditions of eyes open and eyes closed, respectively. The interval between consecutive tests is randomized and controlled by the computer. Consequently, participants cannot predict the timing, direction, or magnitude of the next platform movement.

### 2.4 Experimental procedure

The study employed a single-blind, randomized crossover design, in which session order was random and sessions were separated by 3–7 days. The number “1,” “2,” “3,” and “4” represents the right, left, bilateral and sham stimuli, respectively. The test order of each subject was randomly sorted by a professional statistical expert. The order of stimulation sessions was counterbalanced across participants. All electrodes were worn (P3, P4, PZ, P7, P8, C3, C4, O1, O2) during each stimulation, ensuring that participants remained blinded to the details of each stimulation type. Each session consisted of SOT and MCT under eyes open and eyes closed conditions, all before and immediately after either real or sham stimulation. Subjects were instructed to refrain from consuming alcohol or coffee within 24 h prior to the start of the experiment. Throughout the experiment, the laboratory maintained a quiet environment, ensuring participants remained vigilant, refrained from engaging in conversations with others, and abstained from using their phones. If the participants experienced any discomfort, the experiment was terminated. The duration of each session tasks was approximately 1.5 h.

### 2.5 Data analysis methods

Equilibrium score (ES), composite score (CS) and sensory score (VIS, SOM, VEST and PRE) serves as the primary outcome of the SOT. An equilibrium score is generated under each testing condition, while CS is calculated based on scores from all conditions. The higher the score, the better the balance performance. The CS is the predominant indicator utilized in the SOT (Zhou et al., 2025). Sensory scores are calculated based on the ES, following the formula provided, reflecting the capability of sensory system to maintain balance. PREF represents the capacity to counteract visual interference for the maintenance of postural stability.


SOM=SOT2/SOT1

VIS=SOT4/SOT1

VEST=SOT5/SOT1

PREF=(SOT3+SOT6)/(SOT2+SOT5)

The latency and response strength serves as the primary outcome of the MCT. The latency records the time from the onset of perturbation to the reaction of each leg. The latency under different directions and magnitude of platform movement is obtained by averaging the reaction time of both legs. The composite latency is obtained by averaging the reaction times in forward and backward translations at three different magnitude. The response strength quantifies the force applied to the platform, with larger numbers indicating a greater force being applied.

**Figure 2:**
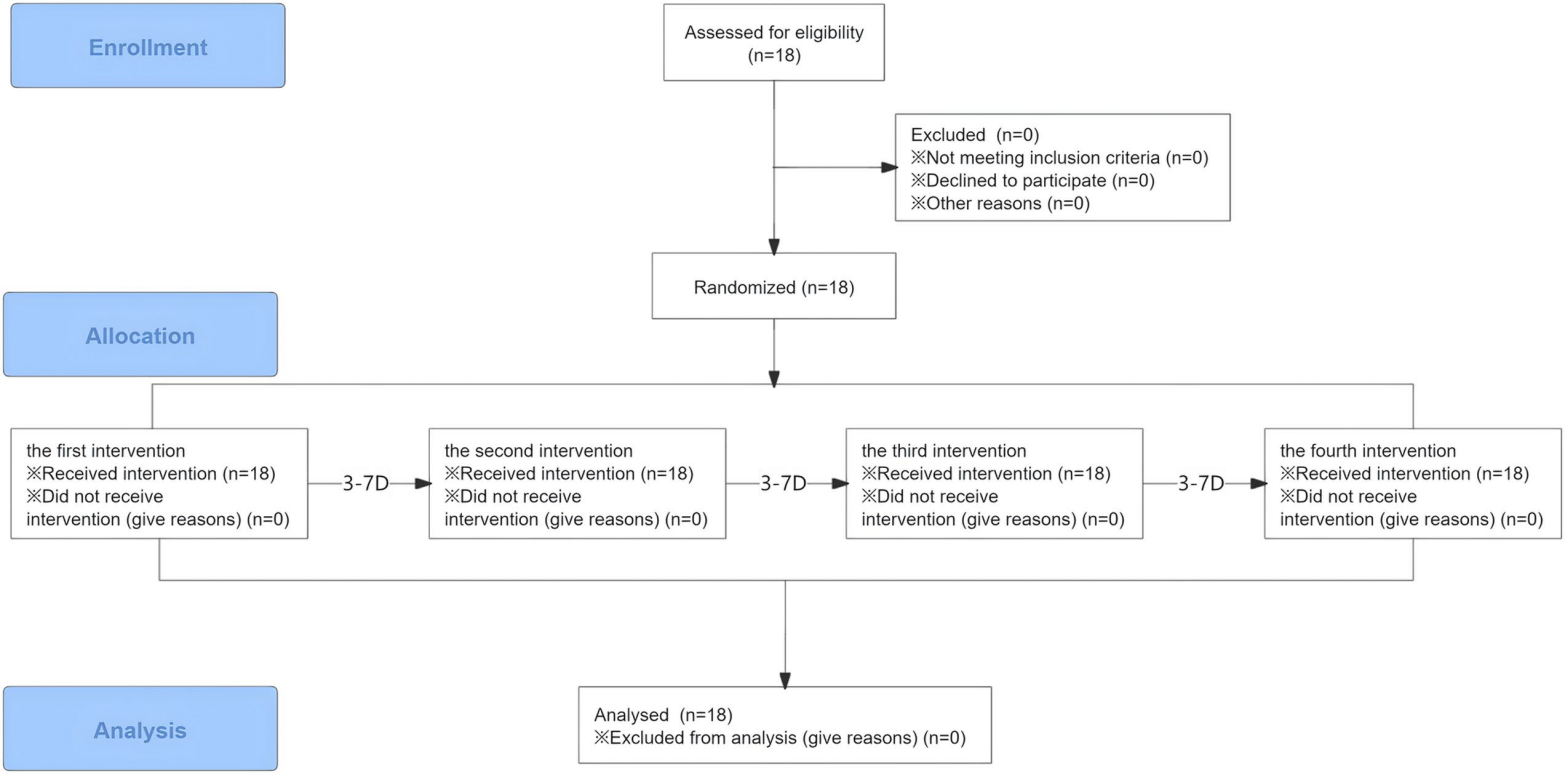
Flow diagram.

All data were analyzed by SPSS 25.0 and presented as Mean ± SD. Using Shapiro–Wilk tests to evaluate the normal distribution of the data. Two-way repeated measures analysis of variance (ANOVA) was used to analyze the dependent variables. Bonferroni’s *post hoc* tests were used in case of significant ANOVA results. The significance threshold was set at *α* = 0.05 (Figure 2).

## 3 Results

No adverse reactions or events occurred during the entire experiment, such as skin redness, headache, dizziness or other events.

### 3.1 ES CS and sensory score

The four experimental sessions showed no significant differences in ES, CS, SOM, VIS, VEST, and PREF at baseline (all values of *p* > 0.05). The main effect of time is significant different for ES1 [*F_(1, 17)_* = 11.328, *p* = 0.004, *η*_p_^2^ = 0.4], post-hoc comparisons revealed that ES1 decreased significantly only after right (*p* = 0.011) and sham (*p* = 0.035) stimulation. For SOT2-6, there are no significant main effects of time, group, or interaction effects. The results of two-way repeated measures ANOVAs of SOT are presented in Table 1. The means (SD) of SOT are presented in Table 2.

**Table 1:** Results of two-way repeated-measures ANOVAs of SOT.

Parameter	Condition	Factor	df	*F*	*p*	*η*_p_^2^
Equilibrium score	ES1	Time	1,17	11.328	0.004*	0.400
		Session	3,15	1.102	0.379	0.181
		Time × session	3,15	0.593	0.629	0.106
	ES2	Time	1,17	3.064	0.098	0.153
		Session	3,15	0.74	0.544	0.129
		Time × session	3,15	1.507	0.253	0.232
	ES3	Time	1,17	4.419	0.051	0.206
		Session	3,15	0.432	0.733	0.079
		Time × session	3,15	0.452	0.720	0.083
	ES4	Time	1,17	0.572	0.460	0.033
		Session	3,15	0.409	0.749	0.076
		Time × session	3,15	0.998	0.421	0.166
	ES5	Time	1,17	0.337	0.569	0.019
		Session	3,15	0.525	0.672	0.095
		Time × session	3,15	1.427	0.274	0.222
	ES6	Time	1,17	0.774	0.391	0.044
		Session	3,15	0.196	0.898	0.038
		Time × session	3,15	1.638	0.223	0.247
	CS	Time	1,17	0.284	0.601	0.016
		Session	3,15	0.142	0.933	0.028
		Time × session	3,15	1.381	0.287	0.216
Sensory score	SOM	Time	1,17	1.123	0.304	0.062
		Session	3,15	2.977	0.065	0.373
		Time × session	3,15	0.123	0.945	0.024
	VIS	Time	1,17	0.006	0.939	0.001
		Session	3,15	0.326	0.807	0.061
		Time × session	3,15	0.854	0.486	0.146
	VEST	Time	1,17	2.221	0.154	0.116
		Session	3,15	0.163	0.920	0.032
		Time × session	3,15	1.421	0.276	0.221
	PREF	Time	1,17	3.427	0.082	0.168
		Session	3,15	0.667	0.585	0.118
		Time × session	3,15	1.259	0.324	0.201

*p < 0.05; ES, equilibrium score; CS, composite score; VIS, visual score; SOM, somatosensory score; VEST, vestibular score; PREF, visual preference score.

**Table 2:** SOT scores before and after HD-tDCS or sham intervention (Mean±SD).

Parameter	Condition	Time	R-HD-tDCS	L-HD-tDCS	B-HD-tDCS	S-HD-tDCS
Equilibrium score	ES1	Pre	93.13 ± 1.90	91.67 ± 4.04	92.37 ± 2.54	92.94 ± 2.32
		Post	91.52 ± 2.55*	90.22 ± 4.04	91.65 ± 2.98	90.96 ± 4.15*
	ES2	Pre	92.37 ± 2.43	91.70 ± 3.16	92.13 ± 2.85	91.80 ± 2.34
		Post	90.89 ± 3.79	91.24 ± 3.06	92.20 ± 2.25	89.95 ± 5.06
	ES3	Pre	92.89 ± 1.88	92.30 ± 2.69	92.70 ± 2.26	92.35 ± 2.48
		Post	91.35 ± 3.22	91.56 ± 3.39	92.02 ± 3.34	91.28 ± 3.93
	ES4	Pre	72.20 ± 10.87	70.17 ± 10.88	71.65 ± 15.24	73.57 ± 13.38
		Post	71.43 ± 12.46	72.19 ± 15.04	68.37 ± 16.26	71.67 ± 11.54
	ES5	Pre	67.31 ± 11.61	64.63 ± 11.77	67.17 ± 14.20	68.44 ± 11.65
		Post	69.91 ± 10.11	68.15 ± 10.07	66.20 ± 14.26	66.67 ± 15.40
	ES6	Pre	66.00 ± 12.22	64.19 ± 13.21	62.26 ± 16.21	64.50 ± 14.74
		Post	61.65 ± 16.20	65.41 ± 14.90	62.26 ± 19.52	63.69 ± 16.14
	CS	Pre	77.22 ± 7.08	75.44 ± 7.10	76.11 ± 9.07	76.72 ± 8.34
		Post	76.11 ± 7.71	76.67 ± 8.32	75.06 ± 10.36	75.89 ± 9.44
Sensory score	VIS	Pre	77.39 ± 12.01	76.56 ± 11.67	77.67 ± 16.94	79.28 ± 14.55
		Post	78.06 ± 13.41	79.83 ± 15.79	74.72 ± 17.89	78.67 ± 11.28
	SOM	Pre	99.00 ± 2.40	100.06 ± 3.61	99.78 ± 3.64	98.89 ± 2.52
		Post	99.33 ± 3.48	101.06 ± 3.02	100.78 ± 3.64	99.00 ± 4.77
	VEST	Pre	72.11 ± 12.60	70.33 ± 12.12	72.44 ± 15.45	72.72 ± 14.88
		Post	76.33 ± 10.71	75.50 ± 10.74	72.28 ± 15.40	72.94 ± 15.37
	PREF	Pre	99.61 ± 5.08	100.44 ± 5.80	97.50 ± 6.55	97.78 ± 7.51
		Post	95.39 ± 9.94	98.39 ± 6.06	97.17 ± 6.75	99.17 ± 5.34

*p < 0.05, Significant changes from pre-test to post-test; R-HD-tDCS, right HD-tDCS; L-HD-tDCS, left HD-tDCS; B-HD-tDCS, bilateral HD-tDCS; S-HD-tDCS, sham stimulation; ES, equilibrium score; CS, composite score; VIS, visual score; SOM, somatosensory score; VEST, vestibular score; PREF, visual preference score.

### 3.2 Latency, response strength

The four experimental sessions showed no significant differences in latency and response strength under eyes open and eyes closed conditions at baseline (all values of *p* > 0.05).

#### 3.2.1 Latency

For eyes open, the main effect of time is significant for composite latency [*F_(1, 17)_* = 17.857, *p* = 0.001, *η*_p_^2^ = 0.512], post-hoc comparisons revealed that composite latency increased significantly after right (*p* = 0.025) and bilateral (*p* = 0.004) stimulation. The main effect of time is significant in medium and large backward translation [medium backward: *F_(1, 17)_* = 26.378, *p* < 0.001, *η*_p_^2^ = 0.608; large backward: *F_(1, 17)_* = 8.064, *p* < 0.001, *η*_p_^2^ = 0.322], post-hoc comparisons revealed that there is no significant change after true and sham stimulation (*p* > 0.05).

For eyes closed, the main effect of time is significant in large forward translation [*F_(1, 17)_* = 4.450, *p* = 0.032, *η*_p_^2^ = 0.243], post-hoc comparisons revealed that latency increased significantly after left (*p* = 0.003) and bilateral (*p* = 0.04) stimulation. The results of two-way repeated-measures ANOVAs of latency are presented in Table 3. The means (SD) of latency are presented in Table 4.

**Table 3:** Results of two-way repeated-measures ANOVAs of latency under eyes open and eyes closed.

Parameter	Condition			Factor	df	*F*	*p*	*η*_p_^2^
Latency	EO	Composite score		Time	1,17	17.857	0.001*	0.512
				Session	1.538,26.140	0.117	0.838	0.007
				Time × session	3,15	0.314	0.815	0.059
		Backward	Medium	Time	1,17	26.378	<0.001*	0.608
				Session	1.900,32.307	0.436	0.640	0.025
				Time × session	3,15	0.711	0.560	0.125
			Large	Time	1,17	8.064	0.011*	0.322
				Session	2.094,35.605	0.567	0.580	0.032
				Time × session	1.732,29.440	0.027	0.970	0.002
		Forward	Medium	Time	1,17	1.234	0.282	0.068
				Session	1.697,28.855	0.327	0.688	0.019
				Time × session	3,15	0.389	0.763	0.072
			Large	Time	1,17	1.489	0.239	0.081
				Session	3,15	0.199	0.896	0.038
				Time × session	1.716,29.173	0.358	0.690	0.021
	EC	Composite score		Time	1,17	3.094	0.097	0.154
				Session	3,15	0.047	0.986	0.009
				Time × session	1.498,25.459	0.085	0.866	0.005
		Backward	Medium	Time	1,17	1.435	0.247	0.078
				Session	3,15	0.768	0.530	0.133
				Time × session	3,15	0.233	0.872	0.044
			Large	Time	1,17	2.719	0.118	0.138
				Session	3,15	0.066	0.977	0.013
				Time × session	3,15	2.942	0.067	0.370
		Forward	Medium	Time	1,17	0.093	0.764	0.005
				Session	1.993,33.877	0.088	0.965	0.017
				Time × session	2.014,34.238	0.504	0.686	0.091
			Large	Time	1,17	5.450	0.032*	0.243
				Session	3,15	0.916	0.457	0.155
				Time × session	1.628,27.677	0.817	0.438	0.046

* *p* < 0.05; EO, eyes open; EC, eyes closed.

**Table 4:** Latency under eyes open and eyes closed before and after HD-tDCS or sham intervention (Mean±SD).

Parameter	Condition			Time	R-HD-tDCS	L-HD-tDCS	B-HD-tDCS	S-HD-tDCS
Latency	EO	Composite score		Pre	129.78 ± 7.56	130.17 ± 6.38	130.06 ± 7.29	130.78 ± 7.33
				Post	132.28 ± 8.68*	132.11 ± 6.69	133.11 ± 6.58*	132.28 ± 6.88
		Backward	Medium	Pre	128.97 ± 7.51	128.61 ± 6.11	129.22 ± 7.95	128.36 ± 7.27
				Post	131.28 ± 8.55	130.39 ± 5.81	133.44 ± 8.55	132.03 ± 7.30
			Large	Pre	126.31 ± 4.54	127.44 ± 5.97	127.22 ± 7.17	125.83 ± 3.87
				Post	128.83 ± 5.34	130.08 ± 8.22	130.03 ± 4.65	128.94 ± 7.20
		Forward	Medium	Pre	135.31 ± 12.18	133.39 ± 10.02	133.11 ± 9.87	136.17 ± 12.99
				Post	136.25 ± 12.46	134.83 ± 11.31	136.47 ± 12.61	135.94 ± 12.09
			Large	Pre	129.06 ± 10.49	131.25 ± 9.52	130.75 ± 10.76	132.28 ± 13.19
				Post	133.06 ± 14.72	133.06 ± 12.06	132.08 ± 10.05	132.28 ± 11.23
	EC	Composite score		Pre	130.50 ± 7.85	130.56 ± 8.15	130.11 ± 7.32	130.72 ± 8.69
				Post	131.17 ± 8.71	131.61 ± 6.78	131.89 ± 6.67	132.11 ± 6.82
		Backward	Medium	Pre	129.78 ± 9.40	130.42 ± 10.50	128.33 ± 6.99	130.42 ± 8.63
				Post	131.17 ± 10.34	132.03 ± 7.64	129.94 ± 7.54	130.22 ± 5.90
			Large	Pre	128.25 ± 7.00	128.69 ± 6.83	127.56 ± 5.17	127.56 ± 4.58
				Post	129.03 ± 5.65	128.36 ± 4.50	130.44 ± 7.47	130.56 ± 6.42
		Forward	Medium	Pre	132.08 ± 10.09	132.50 ± 12.35	134.67 ± 10.78	134.53 ± 18.50
				Post	134.92 ± 15.03	133.67 ± 10.44	133.83 ± 12.21	136.00 ± 13.30
			Large	Pre	128.61 ± 9.89	127.86 ± 8.62	129.92 ± 11.86	130.11 ± 12.05
				Post	128.58 ± 11.04	132.08 ± 11.65*	133.14 ± 9.92*	131.31 ± 9.69

*p < 0.05, Significant changes from pre-test to post-test; R-HD-tDCS, right HD-tDCS; L-HD-tDCS, left HD-tDCS; B-HD-tDCS, bilateral HD-tDCS; S-HD-tDCS, sham stimulation; EO, eyes open; EC, eyes closed.

#### 3.2.2 Response strength

For eyes open, in forward and backward translations at three different magnitudes, the main effect of time is significant. However there are no significant main effect of session and the interaction effect.

For eyes closed, in forward and backward translations at three different magnitudes, the main effect of time is significant. Post-hoc comparisons revealed that for forward translation at three different magnitudes, response strength decreased significantly after only bilateral stimulation (*p* < 0.05). The results of two-way repeated-measures ANOVAs of response strength are presented in Table 5. The means (SD) of response strength are presented in Table 6.

**Table 5:** Results of two-way repeated-measures ANOVAs of response strength under eyes open and eyes closed.

Parameter	Condition			Factor	df	*F*	*p*	*η*_p_^2^
Response strength	EO	Backward	Small	Time	1,17	46.985	<0.001*	0.734
				Session	3,15	0.867	0.480	0.148
				Time × session	2.168,37.160	0.272	0.783	0.016
			Medium	Time	1,17	69.068	<0.001*	0.802
				Session	3,15	1.273	0.319	0.203
				Time × session	1.982,33.687	0.626	0.539	0.036
			Large	Time	1,17	37.947	<0.001*	0.691
				Session	3,15	1.904	0.172	0.276
				Time × session	1.945,33.059	1.065	0.355	0.059
		Forward	Small	Time	1,17	26.882	<0.001*	0.613
				Session	1.819,30.902	0.229	0.875	0.044
				Time × session	1.950,33.157	0.803	0.454	0.045
			Medium	Time	1,17	14.634	0.001*	0.463
				Session	3,15	0.698	0.568	0.122
				Time × session	1.762,29.948	1.383	0.265	0.075
			Large	Time	1,17	17.671	0.001*	0.510
				Session	1.999,33.983	1.247	0.300	0.068
				Time × session	1.412,24.001	0.672	0.470	0.038
	EC	Backward	Small	Time	1,17	9.660	0.006*	0.362
				Session	1.970,33.4491	0.489	0.615	0.028
				Time × session	1.976,33.587	0.129	0.899	0.008
			Medium	Time	1,17	10.524	0.005*	0.382
				Session	1.974,33.560	0.450	0.639	0.026
				Time × session	1.680,28.559	0.865	0.415	0.048
			Large	Time	1,17	13.078	0.002*	0.435
				Session	3,15	2.183	0.132	0.304
				Time × session	1.759,29.903	0.191	0.800	0.011
		Forward	Small	Time	1,17	5.557	0.031*	0.246
				Session	3,15	0.449	0.722	0.082
				Time × session	1.532,26.051	0.171	0.787	0.010
			Medium	Time	1,17	5.830	0.027*	0.255
				Session	3,15	1.177	0.352	0.191
				Time × session	1.772,30.118	0.343	0.687	0.020
			Large	Time	1,17	7.348	0.015*	0.302
				Session	3,15	1.272	0.320	0.203
				Time × session	1.426,24.235	0.442	0.724	0.025

*p < 0.05; EO, eyes open; EC, eyes closed.

**Table 6:** Response strength under eyes open and eyes closed before and after HD-tDCS or sham intervention (Mean±SD).

Parameter	Condition			Time	R-HD-tDCS	L-HD-tDCS	B-HD-tDCS	S-HD-tDCS
Response strength	EO	Backward	Small	Pre	3.47 ± 1.97	3.19 ± 1.48	2.83 ± 1.29	3.06 ± 1.46
				Post	2.47 ± 1.27*	2.19 ± 0.96*	2.19 ± 1.14*	2.11 ± 1.02*
			Medium	Pre	5.58 ± 1.76	5.25 ± 2.02	5.08 ± 1.66	5.03 ± 1.61
				Post	4.39 ± 1.66*	3.97 ± 1.75*	3.61 ± 1.28*	4.14 ± 1.61*
			Large	Pre	5.56 ± 1.71	5.44 ± 1.71	5.31 ± 1.81	4.81 ± 1.30
				Post	4.67 ± 1.33	4.56 ± 1.16*	3.97 ± 1.08*	4.36 ± 1.53*
		Forward	Small	Pre	3.67 ± 1.95	3.17 ± 1.63	3.53 ± 1.56	3.47 ± 1.92
				post	2.58 ± 1.36*	2.69 ± 1.31*	2.42 ± 1.49*	2.67 ± 1.35*
			Medium	Pre	5.39 ± 2.16	4.58 ± 1.63	4.64 ± 1.70	4.47 ± 1.69
				Post	3.94 ± 1.54*	3.97 ± 1.16*	3.69 ± 1.68*	3.94 ± 1.47
			Large	Pre	5.72 ± 2.41	4.97 ± 1.59	5.31 ± 1.56	4.89 ± 1.66
				Post	4.50 ± 1.54	4.39 ± 1.17*	4.25 ± 1.23*	4.31 ± 1.62*
	EC	Backward	Small	pre	2.97 ± 1.46	2.69 ± 1.50	2.78 ± 1.49	2.77 ± 1.51
				Post	2.47 ± 1.23	2.39 ± 0.99	2.22 ± 1.03*	2.31 ± 1.13
			Medium	Pre	4.64 ± 1.92	4.42 ± 1.56	4.81 ± 1.76	4.39 ± 1.56
				post	4.28 ± 1.56	4.00 ± 1.58	3.81 ± 1.39*	4.00 ± 1.75*
			Large	Pre	5.61 ± 1.65	4.94 ± 1.72	5.00 ± 1.40	4.97 ± 1.76
				Post	4.94 ± 1.63	4.25 ± 1.19*	4.08 ± 1.44*	4.36 ± 1.30*
		Forward	Small	Pre	3.44 ± 1.62	3.06 ± 1.58	3.14 ± 1.73	3.32 ± 2.07
				Post	3.03 ± 1.60	2.78 ± 1.39	2.58 ± 1.33*	2.64 ± 1.37
			Medium	Pre	5.22 ± 2.01	4.25 ± 1.80	4.44 ± 1.78	4.53 ± 2.15
				Post	4.53 ± 1.72	4.06 ± 1.47	3.92 ± 1.24*	4.06 ± 1.36
			Large	Pre	5.78 ± 2.16	4.89 ± 1.76	5.11 ± 1.75	5.06 ± 1.65
				Post	4.86 ± 1.59	4.47 ± 1.24	4.61 ± 1.61*	4.72 ± 1.29

*p < 0.05, Significant changes from pre-test to post-test; R-HD-tDCS, right HD-tDCS; L-HD-tDCS, left HD-tDCS; B-HD-tDCS, bilateral HD-tDCS; S-HD-tDCS, sham stimulation; EO, eyes open; EC, eyes closed.

## 4 Discussion

The current study observed that enhancing the excitability of the right parietal lobes under eyes open conditions and the left parietal lobes under eyes closed conditions using HD-tDCS led to a prolongation of the latency of automatic postural responses. These findings demonstrate that the right parietal lobe is involved in modulating automatic responses when visual information is input normally, while the left parietal lobe is involved when visual information is shielded. When the platform was translated forward at three different magnitudes under eyes closed conditions, response strength decreased only after bilateral stimulation. This suggests that bilateral parietal HD-tDCS is beneficial for enhancing the ability of postural responses. The study did not find that unilateral or bilateral parietal lobes HD-tDCS could improve the ability of vision, somatosensory and vestibular system to maintain postural control in young healthy adults.

Rapid response to external interference is necessary in daily life to maintain stability. For example, it is important to take corresponding balance strategies in time to prevent falls while walking on a wet or icy road. Postural responses can be categorized into two phases: an early phase, from the onset of perturbation to the initial rapid reaction, known as the automatic postural response; and a later phase, involving the establishment of a new steady-state posture (Honeine et al., 2015). Early studies have suggested that postural response is mainly regulated at subcortical level (Sherrington, 1910). Currently, an increasing number of studies have shown that the cerebral cortex also participates in modulating postural responses (Bolton, 2015; Barollo et al., 2022; Jacobs and Horak, 2007). It is primarily involved in the later stage, optimizing responses to adapt to the surrounding environment and meet individual goals (Jacobs and Horak, 2007; Maki and McIlroy, 2007). Meanwhile, the early stage, which is highly automated, is controlled by the subcortical level (Honeine et al., 2015).

J. V. Jacobs had studied the early and late phase of latency. The authors believe that brainstem circuits initiate an automatic response, which is subsequently optimized by cortical circuits during later phases. The cerebral cortex directly regulates posture responses through the corticospinal loop, leading to a longer latency period. Alternatively, it can indirectly shorten the latency by initiating coordination through the brainstem (Jacobs and Horak, 2007). The increased excitability of parietal lobe directly correlates with its heightened involvement in the postural response.

Studies suggest that the cerebellar-cortical loop is accountable for adapting postural responses based on prior experience (Thach and Bastian, 2004), while the basal ganglia-cortical loop is accountable for pre-selecting and optimizing postural responses based on current context (Takakusaki et al., 2004; Grillner et al., 2005). Increased cortical involvement in the early stage may facilitate interaction between the cortex and subcortical structures, potentially benefiting later-stage adjustments. This hypothesis is consistent with previous research findings. Motor cortex (C3/C4) tDCS activates postural synergies involving brainstem networks via descending projections (Nonnekes et al., 2014). Meanwhile, brainstem networks project to the cortical cortex, expediting cortical processing (Sibley et al., 2014; Jones, 2008). These result suggest that increasing cortical excitability can promote its interaction with subcortical structures.

While this study interprets prolonged latency as indicative of heightened cortical engagement, the previous studies suggest that such electrophysiological delays may reflect compromised neural processing efficiency or subclinical impairment, potentially associated with postural instability and increased fall risk in clinical populations (Lee et al., 2020). Comprehensive investigation of postural reactions necessitates moving beyond exclusive focus on latency to quantify the total stabilization period, defined as the temporal interval from perturbation onset to complete balance recovery. Biomechanical investigations have revealed that athletes with concussion histories exhibit shorter step latency compared to controls, yet paradoxically demonstrate prolonged time-to-stabilization (Monoli et al., 2025). A limitation of this study is reliance on latency to assess postural responses, therefore future research should integrate evaluation of total response time. The observed prolongation of automatic postural responses in this study suggests that enhanced cortical engagement facilitates cortico-subcortical network interactions to optimize posture response. Moreover, the outcome of response strength in the present study further support the hypothesis.

Previous studies have concluded that the MCT has no learning effect (Hill et al., 2018; Hale et al., 2009), which generates the two sub-strategies of postural control: ankle strategy and hip-knee strategy (Pollock et al., 2000). Repeated exposure to MCT allows CNS to select a more effective motor strategy, transitioning from a hip-knee to an ankle strategy (Hill et al., 2018). This change may result in a decrease in response strength. Under moderate disturbance, the default strategy is the ankle strategy. As the disturbance difficulty increases, there is a shift from the ankle strategy to the hip strategy. The participants in the previous study reported that maintaining balance is more difficult when the platform moves forward compared to moving backward (Hill et al., 2018). The activation levels of lower extremity muscles increase with the perturbation magnitude during the MCT (Hill et al., 2018). Meanwhile, in the case of closed eyes, maintaining posture becomes more challenging, resulting in increased activation of muscles. When the platform moves forward under eyes closed conditions, bilateral parietal stimulation may increase the involvement of the cerebral cortex, promoting coordination between the parietal lobe and subcortical structures. These resulted in the CNS selecting a more effective balance strategy and reducing response strength. Rapid adaptation of the neuromuscular system’s reactive to novel perturbations decreases the possibility of improper motor response (Hill et al., 2018). Bilateral tDCS on PPC enhances postural adaptation following tilt in healthy young adults (Young et al., 2020). Parietal tDCS may function by enhancing the rapid adaptation to facilitate optimized postural responses, thereby reducing response strength.

Postural control is predominantly dependent on the integration of sensory information derived from visual, somatosensory, and vestibular inputs. The right hemisphere-driven processing of multisensory information in the posterior parietal cortex (Arshad et al., 2014; Pisella et al., 2011; Convento et al., 2018) may account for the prolonged latency observed with right stimulation during eyes open condition, while the left stimulation’s lack of effect suggests a different mechanism at play. In daily life and during physical activities, individuals are exposed to constantly changing visual environments, requiring them to direct their attention to external control points and maintain neuromuscular to ensure postural stability (Tasci et al., 2024). The visual system provides a fundamental mechanism for coordinating, regulating, and controlling movement, while also managing interactions within the environment (Unell et al., 2021). Spatial reference frames of the body within the environment affects posture reactions. Self-centered spatial maps must be constantly updated when individuals are disturbed, enabling compensatory movements to be rapidly to executed to maintain balance (Maki and McIlroy, 2007). Spatial remapping, updating information across eyes movements, is an important mechanism for trans-saccadic perception, which is the process of integrating the content of individual fixations over space and time into a stable, internal representation of the environment (Prime et al., 2011). The right parietal lobe primarily governs spatial navigation and trans-saccadic perception (Pisella et al., 2011; Ten Brink et al., 2019). The latency remained unchanged during right stimulation under eyes closed conditions, further suggesting that the right parietal lobe primarily processes visual information during postural reactions.

Proprioception (the sense of body position and movement) includes signals from mechanoreceptors (proprioceptors) located in muscles, tendons, and joint capsules. Proprioception plays a critical role in constantly updating information on body segment positions, detecting body sway and monitoring changes in muscle length, particularly surrounding the ankle joint, to maintain steady upright standing (Henry and Baudry, 2019). The vestibular organs, located within the inner ear, consist of bilateral otoliths and three semicircular canals, which detect linear and angular accelerations of the head. Vestibular signals provide real-time environmental information in both egocentric and allocentric reference frames to ensure postural stability (Xie et al., 2024). The left posterior parietal cortex can be activated by somatosensory input, such as light touch from an external stable reference frame (Ishigaki et al., 2016b), as well as by vestibular inputs, like caloric stimulation or galvanic vestibular stimulation (Bense et al., 2001). In a previous study employing repetitive transcranial magnetic stimulation (rTMS) to attenuate the function of the left posterior parietal cortex, the inhibition in cortical function was only observed under the eyes closed condition. Specifically, when participants had their eyes closed, those in the stimulation group exhibited decreased stability and diminished proprioception in both the ankle and knee joints. Moreover, they displayed symptoms akin to sensory ataxia (Bertuccelli et al., 2024). Research indicates that the left parietal lobe primarily processes proprioception and vestibular sensation during posture control (Ishigaki et al., 2016b; Oka et al., 2022). Blocking of visual information leads to reliance on proprioception and vestibular sensation for posture control. Participants exhibit a preference for proprioception over vestibular sensation during the horizontal translation of the support surface (Akçay et al., 2021). MCT is assessed through forward and backward movements on a platform. These findings can explain why left stimulation prolonged latency just under the eyes closed condition. In summary, the parietal lobe shows lateralized roles in postural control: the right side processes visual information, while the left side handles somatosensory input, aligning with prior research.

Perhaps because the subjects in the present study did not have any sensory information processing issues, no significant differences were observed after intervention in the ability of each sensory system to maintain balance.

The results of previous study are consistent with the present. Using a sway-referencing paradigm to assess sensory reweighing processes, the previous study explored the effect of M1 tDCS on postural control in healthy older and young adults (Craig and Doumas, 2017). Many prior investigations into the influence of tDCS on postural control have yielded significant outcomes, focusing on individuals with clinical ailments (Benninger et al., 2010; Kaski et al., 2014; Kaski et al., 2013). A meta-analysis investigating the effects of non-invasive brain stimulation on sensory function in post-stroke patients revealed that tDCS significantly enhances sensory recovery compared to control groups, particularly when targeting the primary motor cortex (M1), primary somatosensory cortex (S1), or combined M1-S1 stimulation (Chen et al., 2023). Combined intervention of bilateral S1 tDCS with sensorimotor training significantly enhanced light touch perception, stereognosis, and proprioception in post-stroke patients (Li et al., 2023). Consequently, the subjects recruited may have influenced outcomes. In future studies, participants with sensory integration deficits could be selected to investigate whether HD-tDCS of the parietal lobe improves the stability-maintaining abilities of the visual, somatosensory, and vestibular systems.

Currently, the effects of parietal tDCS on postural control remain inconclusive. Cathodal tDCS applied to the PPC reduces the influence of light touch on postural control, but does not affect static standing balance (Ishigaki et al., 2016a). However, unilateral parietal cathodal tDCS can impair static standing balance in healthy young individuals (Oka et al., 2022). Previous studies have largely confirmed the role of the parietal lobe in postural control by using cathodal stimulation to attenuate its function. However, whether anodal stimulation of the parietal lobe can enhance static or dynamic postural control remains to be further investigated. Bilateral tDCS on PPC enhances postural adaptation following tilt in healthy young adults. Notably, this improvement is independent of stimulation polarity, as both configurations—anodal stimulation over the left PPC with cathodal placement on the right, and vice versa—demonstrate comparable efficacy in augmenting postural adaptability (Young et al., 2020). Postural responses are regulated through the interaction between cortical and subcortical structures. The prolongation of latency induced by parietal tDCS suggests that tDCS may modulate sensorimotor functions via corticospinal and subcortical pathway. The previous study suggested that increased corticospinal excitability following anodal tDCS may result from activation of voltage-sensitive Ca^2+^ channels, increased influx of Ca^2+^, which subsequently elevates NMDA receptor sensitivity, thereby leading to increased motor evoked potentials (MEPs). Although afferent feedback may regulate pacing during self-paced exercise, enhanced corticospinal excitability did not improve motor performance (Kristiansen et al., 2021). Emerging evidence indicated that corticospinal excitability was suppressed during anodal tDCS, with no persistent modulation observed after stimulation (Takano et al., 2023). Additionally, whether stimulation of different hemispheres would produce different outcomes is also a key focus for future research.

## 5 Study limitations

This study mainly discusses the impact of parietal HD-tDCS on postural control from the perspective of sensory information processing. The indicators involve equilibrium score, composite score, sensory score, latency and response strength under different test conditions, which have certain limitations for the expression of results. The postural response solely evaluated the latency in automatic posture response without assessing the time course of reaction. The analysis of motor strategy primarily relies on reaction strength, overlooking the muscle activation of different strategies. It is important to record electromyography (EMG) of trunk and lower extremity to enhance the precision of conclusions. Matching the difficulty of the test task with the capabilities of the subjects may lead to better-presented test results. The participants included in such experiment should be carefully considered. In future studies, the utilization of functional near-infrared brain imaging and EEG analysis can provide an intuitive understanding of cortical activity during posture control.

## 6 Conclusion

The parietal lobe participates in modulating automatic postural responses. The primary role of the right parietal lobe in postural response is to process visual information, while the left parietal lobe processes somatosensory information.

## Data Availability

The raw data supporting the conclusions of this article will be made available by the authors, without undue reservation.

## Footnotes

^1^ http://www.chictr.org.cn
